# AI-Guided Home-Based Therapy for Convergence Insufficiency: A Comparative Feasibility Study with Pencil Push-Ups

**DOI:** 10.3390/bioengineering13070828

**Published:** 2026-07-17

**Authors:** Ahmad Khatib, Shmuel Raz, Elad Shvartz, Ilan Shimshoni, Haneen Jabaly-Habib

**Affiliations:** 1 Department of Information Systems, University of Haifa, Haifa 3498838, Israel; razshmu@gmail.com (S.R.); ishimshoni@is.haifa.ac.il (I.S.); 2 Department of Ophthalmology, Tzafon Medical Center, Tiberias 1520800, Israel; eladshvartz@gmail.com (E.S.); hjabaly@gmail.com (H.J.-H.); 3 Azrieli Faculty of Medicine, Bar Ilan University, Safed 1311502, Israel

**Keywords:** convergence insufficiency, tele-ophthalmology, ophthalmic AI applications, digital therapeutics, clinical AI integration, eye motility disorders, artificial intelligence

## Abstract

Background: Convergence insufficiency (CI) is a common binocular vision disorder that causes eye strain, headaches, and blurriness. Although pencil push-ups (PPU) are widely used to treat CI, their real-world effectiveness may be limited by inconsistent performance and poor adherence. MobileS (the App) is an AI-based smartphone application that tracks eye movements through the front camera, enabling guided “digital push-ups” with instant visual feedback and automatic performance monitoring. This follow-up study evaluated the feasibility, recorded adherence, and preliminary clinical signals of MobileS as a home-based therapy for CI compared with conventional PPU. Methods: Twenty-five participants with CI were randomly assigned to either the App group (*n* = 12) or the PPU group (*n* = 13) for 8 weeks of home-based training. Both groups performed convergence push-ups—using the app or a pencil. NPC was measured using a RAF ruler at baseline, mid-point, and endpoint. Symptom severity was assessed using the Convergence Insufficiency Symptom Survey (CISS), and adherence was recorded automatically by the app or manually by participants. Results: All 25 randomized participants completed all three NPC assessment points. The App group showed a statistically significant improvement in NPC from baseline to study completion (mean improvement = 2.81 cm, *p* = 0.007), whereas the PPU group showed no significant change. Recorded adherence was higher in the App group compared with the PPU group (90.1% vs. 75.4%, *p* = 0.0069). The App group showed a significant reduction in symptom severity (*p* = 0.049). Between-group differences in symptom change were not statistically significant. Conclusions: MobileS appears feasible as a home-based CI management tool, and was associated with higher recorded adherence and preliminary NPC improvement signals. Larger, adequately powered studies are needed to confirm efficacy, particularly for symptoms, and guide teleophthalmology implementation.

## 1. Introduction

Convergence insufficiency (CI) is a common binocular vision disorder characterized by difficulty maintaining convergence on near targets, leading to symptoms such as eye strain, blurred vision, headaches, and difficulty concentrating [1,2,3,4]. The growing reliance on digital screens has further amplified the clinical relevance of CI, particularly among students and remote workers [5,6,7,8].

Conventional management of CI includes both home- and office-based vision therapy, such as pencil push-ups (PPU), vergence or accommodative therapy, prism correction, and, in severe cases, surgery. Among these, PPU remains the most widely prescribed method due to its simplicity, low cost, and ease of self-administration [9,10]. However, the effectiveness of PPU has been increasingly questioned. Randomized controlled trials have shown that while PPU may yield only modest short-term improvements, it often fails to achieve sustained normalization of the near point of convergence (NPC) or a meaningful reduction in symptoms [11,12,13,14]. These limitations have coincided with the emergence of digital and AI-assisted technologies in ophthalmology.

In CI management, the near point of convergence (NPC) is a widely used, objective marker of convergence performance and is commonly monitored over time to reflect clinical change [1,3]. In home-based regimens, however, clinical benefit depends not only on the prescribed exercise principle but also on how consistently it is performed. Adherence is therefore a critical determinant of outcomes in unsupervised therapy, and improving engagement and consistency remains a central challenge for conventional pencil push-ups [3,10]. Digital, feedback-driven platforms may help address this gap by standardizing practice and supporting sustained participation in daily training.

While augmented reality (AR) and virtual reality (VR) have been explored for ophthalmic rehabilitation, their application to CI remains limited [15]. Reported gains have generally not surpassed those achieved with office-based therapy, and the cost and accessibility of VR systems limit their scalability for widespread home use [16,17], which makes mobile health (mhealth) tools particularly suited to address visual strain associated with prolonged near work initially expressed by increased NPC [18,19,20].

However, a comprehensive analysis of 160 vision-related mobile apps found that the vast majority targeted basic visual functions—such as acuity, colour vision, or field testing—while only a handful addressed binocular parameters, and none offered therapeutic functionality for binocular or accommodative dysfunctions [21]. Similarly, a systematic review of 48 clinically evaluated ophthalmic mobile apps reported that fewer than 5% were designed for therapeutic use, and most lacked clinical validation or professional oversight [22]. These findings indicate a need for further validation of AI-driven platforms capable of extending beyond screening toward autonomous diagnostic and therapeutic applications in binocular vision care.

A recent review of mobile health technologies in ophthalmology identified significant gaps among existing applications—most lacked specialized functionality for binocular or accommodative dysfunction, relied on external hardware, or required clinician validation of AI-generated data. The system proposed by Subbiah et al. [23] specifically aimed to address some of these gaps by introducing a smartphone-based diagnostic tool for NPC measurement, offering an accessible yet clinician-supervised approach to binocular assessment.

Advancing beyond these limitations, we developed MobileS, a smartphone-based application that utilizes artificial intelligence to estimate the near point of convergence (NPC) from live video captured by the device’s front-facing camera [24]. In preliminary validation, the system showed good agreement with standard clinical measurements, supporting its potential use for remote assessment [24].

Our prior work established MobileS as a smartphone-based system for automated NPC estimation and CI screening under controlled conditions [24]. Our broader MobileS research program has also shown that AI-derived graphical outputs from the platform can improve ophthalmologists’ accuracy and consistency in interpreting NPC-related convergence data [25]. The present study extends this line of work from assessment to intervention by exploring whether the same platform can support home-based convergence training in a real-world setting. We compare App-guided therapy with conventional PPUs over an 8-week period, emphasizing applicability outcomes (including adherence and completion) alongside preliminary clinical signals (NPC and symptoms). These findings are intended to facilitate the design of future trials and the potential integration of smartphone-based CI therapy within tele-ophthalmology workflows.

Unlike earlier mobile-based systems—such as those described by Subbiah et al. [23]—which focused solely on accommodative assessment and required clinician verification of AI-generated results, MobileS was designed to operate autonomously. By integrating both measurement and user guidance within a single platform, it enables repeated, self-administered convergence tasks without external equipment or direct supervision.

While the platform was initially intended for diagnostic purposes, its interactive guidance features, reminders, and progress tracking suggest broader therapeutic applicability. Because repeated NPC measurements follow the same fundamental mechanism as conventional PPU—focusing on a near accommodative target and bringing it progressively toward the face until loss of fusion and recovery—the procedure may inherently function as a form of “digital push-up” training. The present study therefore examines the feasibility, recorded adherence, and preliminary clinical signals of MobileS-guided home-based training compared with conventional PPU exercises.

MobileS uses an interactive mechanism—to strengthen convergence performance by reinforcing motivation and precision during exercises. Evidence from teleophthalmology and digital health applications further suggests that platforms incorporating adaptive user feedback may support engagement, and remote monitoring [26,27,28,29].

## 2. Materials and Methods

### 2.1. Study Design

This was a prospective, two-arm, home-based interventional study comparing an AI-based mobile application (MobileS) with conventional PPU exercises for the treatment of CI. The study was approved by the institutional Ethics Committee and conducted in accordance with the Declaration of Helsinki.

Participants with convergence insufficiency (CI) were identified from our previous research dataset and contacted by telephone for preliminary screening, explanation of the study purpose, and confirmation of eligibility and willingness to participate. Eligible individuals were then randomly assigned to either the App group or the PPU group using a computer-based simple randomization procedure without blocking or stratification. The random allocation sequence was generated by the study team before participant assignment. After eligibility was confirmed, participants were assigned to the next allocation in the sequence and were then scheduled for an in-person baseline visit. Participants were not aware of their allocation before assignment.

**Figure 1:**
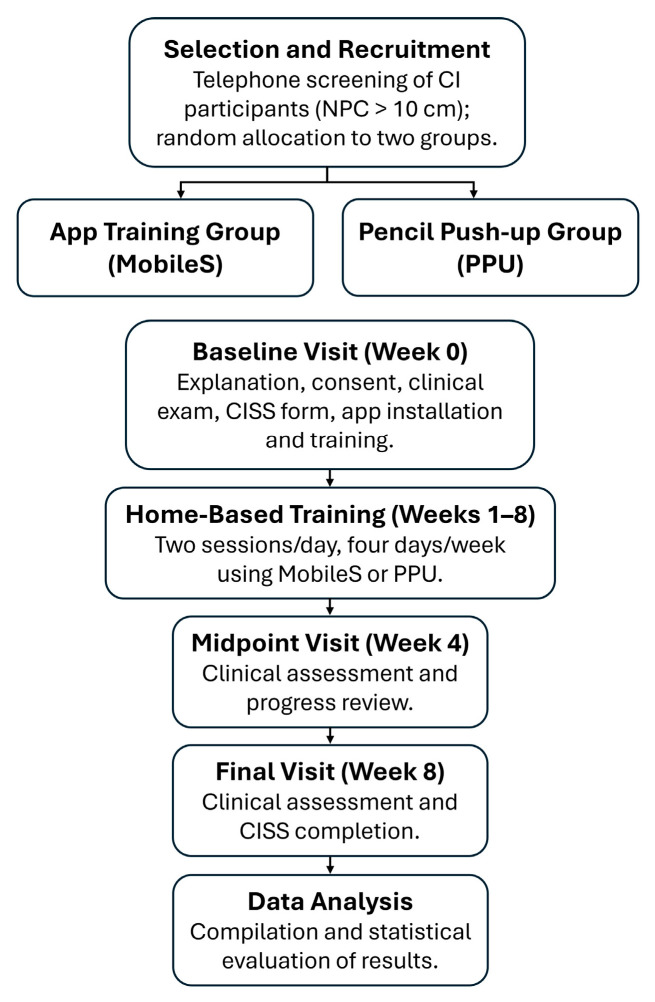
Overview of the study design. Participants with CI were randomly assigned to the App or PPU group and completed baseline, midpoint, and final assessments over an eight-week home-based training period; PPU, pencil push-ups.

This study was designed as a pilot feasibility trial; therefore, no a priori sample-size calculation was performed. The overall study workflow, including participant allocation and follow-up timeline, is illustrated in Figure 1.

### 2.2. Participants

A total of 25 participants with CI (NPC > 10 cm) were recruited and randomly allocated into two intervention groups: the App group (*n* = 12), which used the MobileS application, and the PPU group (*n* = 13), which performed PPU exercises. Inclusion criteria included age 18 years or older, best corrected visual acuity of 6/12 or better in each eye, and the ability to perform exercises independently at home. Exclusion criteria included previous ocular or strabismus surgery, or inability to complete follow-up assessments.

CI eligibility was confirmed by an ophthalmologist during the in-clinic assessment using standard NPC measurement with a RAF ruler. NPC was selected as the primary objective clinical endpoint because it is a widely used marker of convergence performance in CI, can be measured repeatedly using a standard RAF ruler, and directly corresponds to the convergence behavior captured by the MobileS platform. Participants performed both the baseline assessment and the home-training exercises with their habitual near correction when applicable. None of the included participants had received prior CI-directed vision therapy before enrolment.

### 2.3. Intervention Protocol

Participants in both groups followed a structured home-based training regimen over a period of eight weeks. The training frequency was identical for both groups: two sessions per day, four days per week. Each session included ten convergence repetitions.

PPU Group: Participants held a pencil at arm’s length and slowly brought it toward their nose while maintaining single, clear vision. They were instructed to stop when blurriness or diplopia occurred and to repeat the motion ten times per session. Adherence was recorded using a paper log.

App Group: Participants used the MobileS smartphone application (App-guided therapy), which guides convergence exercises through a visual interface and monitors performance using the front-facing camera. As previously described [24], the exercise involves following an on-screen target until the phone reaches the nose. The app provides both written and voice instructions, along with instant visual guidance and session reminders, and progress is automatically logged.

Participants were instructed to perform training while seated, under typical room lighting, and with the target held slightly downward in the natural gaze position, consistent with the in-clinic RAF ruler assessment posture. The operational definitions of a repetition and a session were identical across groups.

**Figure 2:**
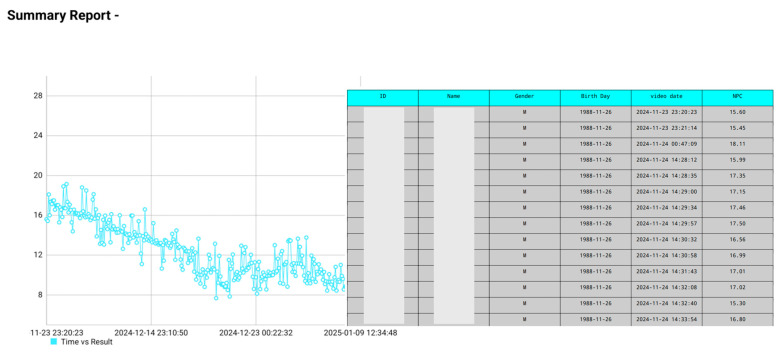
Example of the MobileS application output. The platform automatically records NPC values during home-based training and provides both a graphical summary of progress over time, showing a steady downward trend in NPC values across repeated trials (**left**), and a detailed session log of individual measurements (**right**). This on-screen visual feedback, available directly on participants’ own smartphones, was intended to support engagement and improve adherence throughout the study.

The application was installed directly on each participant’s own smartphone; no dedicated device was provided, and no specialized hardware was required. MobileS is compatible with most standard Android devices, making it widely accessible without additional cost. Following installation, participants received a brief initial training session to ensure proper use, after which they continued independently at home. To promote regular practice, the application displayed instant results, including a graphical summary of progress over time (Figure 2). The longitudinal display of results demonstrated stable measurement patterns across repeated trials under everyday conditions.

### 2.4. Assessment Procedure and Masking

Participants were assessed at three time points: baseline (week 0), midpoint (week 4), and study completion (week 8). At each visit, NPC was measured using a standard RAF ruler.

Participants were not blinded because the interventions were visibly different. NPC outcome assessors were masked to group allocation, and participants were instructed not to disclose their assigned intervention during clinical NPC assessment. Data analysts were not blinded because of the clear differences in data format and intervention-specific adherence records between the MobileS and PPU groups.

In addition, participants completed the Convergence Insufficiency Symptom Survey (CISS) at baseline and at the end of the study. This validated 15-item questionnaire assesses visual discomfort and difficulty during near tasks. Higher scores reflect greater symptom severity [3].

### 2.5. Adherence Monitoring and Statistical Analysis

Adherence was calculated as the percentage of completed sessions out of the total prescribed. In the App group, session completion was recorded automatically by the MobileS application. In the PPU group, session completion was recorded manually by participants using a paper diary. Although the operational definition of a completed session was identical for both interventions, the recording methods differed.

Statistical analysis was performed using a linear mixed-effects model with repeated measures to evaluate time, method, and their interaction. All outcome means reported in the results represent model-estimated least-squares means (LSMeans) derived from this model. Confidence intervals were calculated at the 95% level. Bonferroni adjustment was applied for multiple comparisons, and a significance threshold of *p* < 0.05 was used.

All randomized participants were included in the analysis according to their assigned intervention group. All 25 participants completed baseline, midpoint, and endpoint NPC assessments; therefore, no missing NPC data were present for the primary outcome. All 25 participants also completed baseline and endpoint CISS assessments.

### 2.6. MobileS AI System

MobileS uses the smartphone’s front camera to capture video during each convergence repetition and processes it with an AI model based on MediaPipe’s eye-tracking framework, which employs a single RGB camera to generate a dense 3D mesh of the face and eyes (478 key points). From these data, the system calculates pupillary distance (PD), smartphone-to-eye distance, and eye motion relative to the nasal bridge [30]. A technical overview of the MobileS model is provided in Appendix A, while a full detailed explanation is available in our previous publication that presented its value in diagnosing CI [24]. Session data are transmitted via encrypted channels (HTTPS/TLS) and stored in an access-controlled, HIPAA-compliant cloud environment for secure processing and analysis. To minimize device-related variability in camera resolution and aspect ratio, all recordings are standardized to a fixed square (1:1) format and a uniform input size prior to analysis, ensuring consistent processing across different smartphones. The on-screen fixation target is rendered at a fixed size independent of smartphone screen size to maintain a consistent stimulus during App-guided therapy. In addition, distance estimation accounts for device-specific camera parameters, including focal length (Appendix A), to reduce scaling differences across devices.

Each therapeutic repetition (bringing the phone toward the nose, like PPU) is processed frame-by-frame, so the same AI signals validated for diagnosis provide objective, dynamic convergence metrics and are automatically analyzed and logged (Figure 2).

## 3. Results

### 3.1. Participant Flow and Baseline Characteristics

A total of 25 participants were enrolled and randomized: 12 to the App group and 13 to the PPU group. All randomized participants received the assigned intervention and completed baseline, midpoint, and endpoint NPC assessments. No participants were lost to follow-up, and no participants were excluded after randomization.

Baseline demographic and clinical characteristics are summarized in Table 1. The mean age was 32.50 ± 7.83 years in the App group and 37.54 ± 6.81 years in the PPU group. The App group included 2 females and 10 males, whereas the PPU group included 5 females and 8 males. The observed mean baseline NPC was 15.65 ± 2.89 cm in the App group and 14.92 ± 4.83 cm in the PPU group. Baseline CISS scores were 18.00 ± 10.97 and 20.46 ± 14.32 in the App and PPU groups, respectively.

### 3.2. Recorded Adherence

Participants in the App group had higher recorded training completion than participants in the PPU group. Mean recorded adherence was 90.1% in the App group compared with 75.4% in the PPU group (*p* = 0.0069; Table 2). Because adherence was recorded automatically in the App group and manually by a paper diary in the PPU group, this comparison was interpreted as a recorded adherence measure.

In addition to higher mean recorded adherence, the App group showed a favourable adherence distribution, with a median of 93.8% compared with 76.6% in the PPU group. In the App group, 10 of 12 participants (83.3%) completed ≥80% of prescribed sessions and 7 of 12 (58.3%) completed ≥90%, compared with 5 of 13 (38.5%) and 1 of 13 (7.7%) in the PPU group, respectively. Full completion (100%) was achieved by 3 of 12 App participants (25.0%) versus 1 of 13 PPU participants (7.7%).

### 3.3. Clinical Outcomes—NPC Measurements

NPC was assessed at baseline, midpoint (4 weeks), and endpoint (8 weeks). All 25 randomized participants completed all three NPC assessment points; therefore, the number of participants contributing data at each time point was 12 in the App group and 13 in the PPU group (Table 3).

The repeated-measures mixed-effects model showed a significant time-by-method interaction for NPC (*F*(2,46) = 7.50, *p* = 0.0015), indicating that NPC trajectories differed between intervention groups over time. The model-estimated App-group baseline-to-endpoint improvement was 2.81 cm (SE = 0.75; 95% CI: 1.31 to 4.32; *t*(46) = 3.77; Bonferroni-adjusted *p* = 0.007).

**Figure 3:**
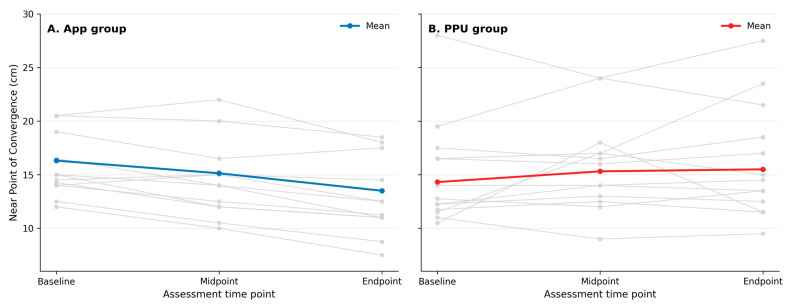
Individual NPC trajectories by intervention group. Thin gray lines represent individual participants, and the colored line represents the group mean. NPC was measured at baseline, midpoint, and endpoint. All randomized participants completed all three assessment points.

Over the 8-week intervention period, the App group demonstrated numerical improvement in NPC across all the assessment time points. In contrast, the PPU group showed no significant change in NPC values over time (Figure 3).

Participant-level trajectories are shown in Figure 3. From baseline to midpoint assessment, 9 participants in the App group and 5 in the PPU group showed improvement. From midpoint to endpoint, 11 participants in the App group improved compared with 6 in the PPU group. When comparing baseline to endpoint, 11 of 12 App participants demonstrated improvement versus only 3 of 13 in the PPU group.

To provide a clinically interpretable threshold-based description, we also examined the number of participants who reached the commonly used clinical NPC threshold of ≤10 cm at the endpoint assessment. At this time point, 2 of 12 App participants reached NPC ≤ 10 cm, compared with 1 of 13 PPU participants.

### 3.4. Symptom Severity—CISS Results

The App group demonstrated a significant within-group reduction in symptom severity from baseline to endpoint (mean change = −5.08; 95% CI: −10.16 to −0.01; *p* = 0.0498), whereas the PPU group showed no significant within-group change (mean change = −2.62; 95% CI: −7.99 to 2.77; *p* = 0.339). However, the direct between-group comparison of CISS change scores did not reach statistical significance (mean difference = −2.47; 95% CI: −9.56 to 4.62; *p* = 0.487).

Exploratory item-level inspection of the CISS suggested broader symptom improvement in the App group across multiple near-work complaints, most notably ocular fatigue or eye discomfort during reading and intermittent blur or words going in and out of focus. In the PPU group, item-level changes were generally smaller, with improvement mainly observed in ocular fatigue.

These symptom findings should be interpreted cautiously because the between-group difference in symptom change was not statistically significant.

## 4. Discussion

This study expands upon our previous research on MobileS, a smartphone-based system initially developed for automated NPC estimation and CI screening. While our earlier work validated its diagnostic performance using front-facing camera tracking of eye convergence behavior, the current investigation evaluated whether the same platform could support home-based convergence training in a real-world, unsupervised setting. Although many mobile tools support home vision therapy by scheduling exercises or timing sessions, MobileS is AI-driven: it computes objective convergence metrics from video, detects NPC break continuously during the therapeutic session itself. This differentiates it from the standard digital therapeutics that lack direct, vision-based and autonomous measurement. This integration of diagnostic and training-related functions may enhance the clinical applicability of the system. To our knowledge, this is among the first pilot studies to compare AI-guided mobile convergence training for CI against the standard PPU in a home-based environment.

NPC improved over time in the App group, while no meaningful change was observed in the PPU group. In the App group, the model-estimated baseline-to-endpoint NPC improvement was 2.81 cm. At the participant level, 11 of 12 App participants showed improvement from baseline to endpoint, compared with 3 of 13 participants in the PPU group. However, normalization was limited: only 2 of 12 App participants reached an endpoint NPC value within the commonly used clinical threshold of ≤10 cm, compared with 1 of 13 participants in the PPU group. These findings suggest a preliminary signal of improved convergence performance, but they do not establish full clinical normalization or definitive treatment efficacy.

These NPC findings are consistent with previous studies reporting limited clinical benefits of PPU alone, particularly regarding long-term symptom relief and objective convergence gains [11,12,13,14]. However, this was a pilot feasibility and proof-of-concept study rather than a definitive efficacy trial. The small sample size, short follow-up period, and baseline imbalance between groups limit the strength of treatment-effect conclusions.

Recorded adherence was higher in the App group than in the PPU group (90.1% vs. 75.4%). This difference may have contributed to the preliminary NPC improvement observed in the App group. Notably, recorded adherence was not only higher on average but also more consistently high across participants in the App group, with a larger proportion completing most prescribed sessions. This pattern may be partly attributed to the platform’s integrated feedback and monitoring features, which address the well-documented challenge of maintaining engagement in unsupervised home-based therapy [21,26]. The application’s accessibility—requiring only a personal smartphone with no additional hardware—and its use of visual progress tracking may also have reduced barriers to regular use.

Nevertheless, adherence results require cautious interpretation because adherence was not recorded using identical methods in both arms. Session completion was recorded automatically in the App group but relied on self-reported paper logs in the PPU group. These methods are not fully equivalent, and self-reporting may be affected by recall bias, social-desirability bias, and reporting bias. Therefore, we considered adherence comparisons as recorded adherence and feasibility indicators rather than as definitive evidence of superior behavioral compliance.

Symptom severity improved within the App group, whereas no significant change was observed in the PPU group. However, the between-group difference in CISS change did not reach statistical significance. Given the modest sample size and short follow-up, symptom-related findings should be interpreted cautiously and confirmed in larger trials with longer follow-up. Exploratory item-level patterns suggested broader improvement across several near-work complaints in the App group, whereas changes in the PPU group appeared smaller and mainly related to ocular fatigue.

From a clinical standpoint, the observed NPC improvement pattern, together with higher recorded adherence and full completion of follow-up assessments, supports the feasibility of MobileS-guided home-based convergence training.

A key implication of this study is the demonstration that MobileS can integrate measurement and guided training functions within a single smartphone-based framework. Most existing applications focus primarily on diagnosis and lack built-in treatment capabilities [21,22]. In contrast, MobileS automates data capture and analysis through AI-driven live eye-tracking and feedback, enabling objective home-based NPC measurements and guided convergence exercises without direct clinician supervision.

A subsequent study in the MobileS research program showed that ophthalmologists interpreted NPC-related convergence data more accurately and consistently when presented with AI-derived graphical outputs than with video recordings alone [25]. Together, these findings suggest that the MobileS platform may support both home-based training by patients and clinical interpretation of convergence data by clinicians.

From a public-health perspective, MobileS may help expand access to binocular vision therapy. Operating entirely on standard smartphones without external hardware, it could be suitable for patients in rural or resource-limited environments, and may support teleophthalmology workflows by enabling remote monitoring of convergence-related data.

This study has several limitations. First, the sample size was small and follow-up was limited to 8 weeks; therefore, the study may be underpowered to detect modest between-group differences, particularly for symptom outcomes. Non-significant between-group findings should be interpreted cautiously.

Second, because simple non-blocked and non-stratified randomization was used, baseline imbalance between groups is possible. Although the App group had a higher observed baseline NPC than the PPU group, this baseline difference should be interpreted descriptively in the context of the small sample size. This imbalance is relevant because participants with higher baseline values may show apparent improvement on repeated testing partly due to regression to the mean, independent of the intervention effect. Therefore, some of the observed NPC improvement in the App group may reflect greater baseline severity rather than the effect of MobileS alone. At the same time, the participant-level trajectories showed that 11 of 12 App participants improved from baseline to endpoint, suggesting that the observed pattern was not driven solely by one or two extreme baseline values. Nevertheless, because the study was small and not stratified by baseline NPC severity, the treatment-related effect cannot be estimated definitively. Future studies should use blocked or stratified randomization according to baseline NPC severity and key demographic variables and consider statistical models adjusted for baseline NPC to reduce the influence of regression-to-the-mean effects.

Third, although NPC was selected as the primary objective clinical endpoint because it is widely used in CI assessment and directly corresponds to the MobileS measurement mechanism, CI is multidimensional. We did not include other standard binocular vision measures, such as accommodative function, fusional vergence, or stereoacuity. Therefore, the present findings should be interpreted as preliminary NPC-related signals rather than a comprehensive assessment of full CI resolution. Future studies should incorporate these outcomes to provide a more precise evaluation of the therapeutic response.

Fourth, self-reported symptoms measured by CISS may introduce subjectivity, and although objective NPC measurements were performed under standardized conditions, variations in home environments could influence training consistency.

Fifth, as noted above, adherence was recorded automatically in the App group but relied on self-reported logs in the PPU group. Future studies may reduce this source of bias by using more comparable adherence monitoring methods across intervention arms, while still preserving the real-world nature of independent home-based training.

Few technical constraints also merit consideration. As a video-based system, MobileS depends on adequate lighting, a stable frontal view, and minimal occlusions, such as hair or spectacle glare. Extreme head positions or rapid device motion can transiently affect landmark stability and tracking precision. The original MediaPipe algorithm attempted to address some of these challenges through built-in stabilization and tracking mechanisms, complemented by on-screen guidance and repetition prompts within the user interface.

In addition, algorithmic factors related to ocular anatomy may influence absolute distance estimation. Subtle limbal irregularities that obscure part of the iris margin, as well as natural interindividual variation in the white-to-white (WTW) corneal diameter used for scaling—typically assumed to be about 11.7 mm—may introduce some variation in absolute distance estimation. Because this variability remains consistent within each subject, it does not affect the longitudinal trend or direction of measured improvement. These factors should nevertheless be considered when generalizing the results to broader populations and environmental conditions.

Larger multicentre trials with longer observation periods are needed to confirm the durability, clinical efficacy, and scalability of these findings. Future versions of the system could incorporate adaptive AI algorithms that adjust exercise intensity, frequency, or feedback based on each user’s ongoing performance patterns—potentially further supporting adherence and individualized home-based training.

## 5. Conclusions

This pilot study suggests that App-guided convergence training using the AI-based MobileS application is feasible as a home-based approach for CI management. Compared with conventional PPU, the App-based approach was associated with higher recorded adherence and preliminary NPC improvement signals over the 8-week training period. Symptom severity improved within the App group; however, between-group differences in symptom change were not statistically significant.

By combining guided “digital push-ups” with automated, objective convergence monitoring using only a standard smartphone, MobileS may help address a key limitation of home therapy, including variable performance, limited feedback, and lack of objective monitoring, while supporting remote follow-up within teleophthalmology workflows.

Larger, adequately powered trials with longer follow-up and more comparable adherence monitoring methods are warranted to confirm clinical efficacy, particularly for symptom outcomes. Future studies should also include additional binocular vision measures, such as accommodative function, fusional vergence, and stereoacuity, and should evaluate longer-term maintenance, usability across diverse devices and settings, and implementation pathways in routine clinical care.

## 6. Patents

This work is protected by U.S. Patent Application CRML-P-047-USP, titled “System and Method for Diagnosis and Treatment of Various Movement Disorders and Diseases of the Eye.” Please note that the patent application is currently pending and has not yet been granted.

## Figures and Tables

**Table 1:** Baseline demographic and clinical characteristics by intervention group.

Variable	App Group (*n* = 12)	PPU Group (*n* = 13)
Age, mean ± SD	32.50 ± 7.83	37.54 ± 6.81
Female, *n* (%)	2 (16.7%)	5 (38.5%)
Male, *n* (%)	10 (83.3%)	8 (61.5%)
Baseline NPC, mean ± SD, cm	15.65 ± 2.89	14.92 ± 4.83
Baseline CISS, mean ± SD	18.00 ± 10.97	20.46 ± 14.32
Emmetropia, *n* (%)	11 (91.7%)	11 (84.6%)
Myopia, *n* (%)	1 (8.3%)	1 (7.7%)
Hyperopia, *n* (%)	0 (0.0%)	0 (0.0%)
Presbyopia, *n* (%)	0 (0.0%)	1 (7.7%)

Values are presented as observed mean ± standard deviation or *n* (%). Refractive status was classified according to spherical equivalent refraction: emmetropia was defined as >−0.50 D and <+0.50 D, myopia as ≤−0.50 D, and hyperopia as ≥+0.50 D. NPC, near point of convergence; CISS, Convergence Insufficiency Symptom Survey; PPU, pencil push-ups; D, diopter.

**Table 2:** Comparison of recorded adherence rates between App and PPU groups.

Methods	*n* (Subjects)	Recorded Adherence (Mean, %)	SE	95% CI
App	12	90.1	3.25	(82.95, 97.25)
PPU	13	75.4	3.71	(67.27, 83.45)

Abbreviations: SE, Standard Error; 95% CI, Confidence Interval.

**Table 3:** Model-estimated NPC values by intervention group and time point.

Methods	*n* (Subjects)	Time Point	NPC (Mean, cm)	95% CI
App	12	Begin	16.31	(14.37, 18.26)
App	12	Med	15.13	(13.18, 17.07)
App	12	End	13.50	(11.55, 15.45)
PPU	13	Begin	14.31	(12.44, 16.18)
PPU	13	Med	15.31	(13.44, 17.18)
PPU	13	End	15.50	(13.63, 17.37)

Values represent least-squares means estimated from the repeated-measures mixed-effects model. The standard error associated with the model-estimated means was 0.97 for the App group and 0.93 for the PPU group across time points. 95% CI, confidence interval; NPC, near point of convergence; PPU, pencil push-ups.

**Figure A1:**
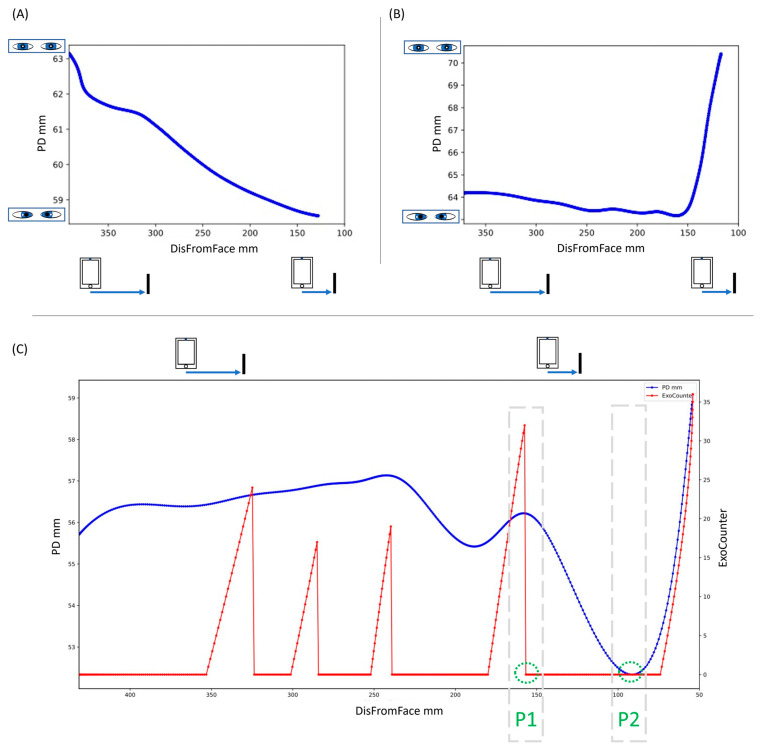
Ref. [24] (**A**) Shows PD variation with phone distance. The subject converges smoothly without interruption; (**B**) depicts CI with a “breaking point” around 16 cm from the face; (**C**) outlines NPC determination, refining PD shift, and integrating ExoCounter by phone distance. P1, last local minimum of the PD; P2, chosen NPC; PD, pupillary distance; DistFromFace, distance from face; ExoCounter, exodeviation episode’s counter; NPC, Near Point of Convergence.

## Data Availability

The data presented in this study are available on request from the corresponding author due to privacy and ethical restrictions. Specifically, video recordings and patient images cannot be shared. However, other anonymized data may be provided upon reasonable request and subject to ethical approval.

## Abbreviations

The following abbreviations are used in this manuscript:

AI	Artificial Intelligence
CI	Convergence Insufficiency
NPC	Near Point of Convergence
MobileS	Mobile application System
HVT	home vision therapy
OBVT	office-based vision therapy
PPU	pencil push-ups therapy
VR	Virtual Reality
CISS	Convergence Insufficiency Symptom Survey
WTW	White-to-white
LSMeans	Least-Squares Means
95% CI	95% Confidence Interval

## Appendix A

The model generates a dense face/eye mesh and localizes the iris centers and limbus contours. From these landmarks, key static parameters are derived, including pupillary distance (PD), white-to-white (WTW) corneal diameter, and smartphone-to-eye distance.

PD (in mm) is computed by scaling the pixel distance between the two iris centers using the iris diameter as a reference. Assuming a constant anatomical iris diameter (WTW) of approximately 11.7 ± 0.5 mm [31,32,33], the following formula is applied:

(A1) $$PD\;=\frac{\left(RightEyeCenter.X-LeftEyeCenter.X\right)\times 11.7}{IrisDiameter}$$

where RightEyeCenter.X and LeftEyeCenter.X denote the horizontal coordinates of the iris centers in pixels, and IrisDiameter is measured in pixels.

Smartphone-to-eye distance is estimated using the camera’s focal length and the detected iris diameter, according to:

(A2) $$DistanceFromCamera\left(d\right)=\frac{FocalLength\left(f\right)\times 11.7\;mm}{IrisDiameter}$$

where (d) is the distance from the eye to the camera in millimeters, (f) is the focal length of the smartphone camera in pixels, and IrisDiameter is again the diameter of the iris in pixels.

Using the device-specific focal length (in pixels) accounts for inter-device FoV differences during distance estimation. Together, PD and smartphone-to-eye distance jointly describe the convergence trajectory as the phone approaches the nose. Tracking the relative motion of each eye to the nasal bridge further enhances stability of the NPC measurement.

During normal convergence, PD and phone-eye distance decrease smoothly ((A) in Figure A1). NPC “break” is identified when PD increases while the phone continues to approach the nose, indicating loss of fusion ((B) in Figure A1); recovery is detected when PD resumes its expected course. The algorithm also flags binocular outward eye drifts (exodeviation episodes; ExoCounter) that often accompany blur or diplopia near the break, and integration with PD dynamics refines NPC detection ((C) in Figure A1).

## References

[1] Gantz L., Stiebel-Kalish H. (2022). Convergence insufficiency: Review of clinical diagnostic signs. J. Optom..

[2] Rouse M.W., Hyman L., Hussein M., Solan H. (1998). Frequency of convergence insufficiency in optometry clinic settings. Optom. Vis. Sci..

[3] Rouse M.W., Borsting E.J., Lynn Mitchell G., Scheiman M., Cotter S.A., Cooper J., Kulp M.T., London R., Wensveen J., Convergence Insufficiency Treatment Trial Group (2004). Validity and reliability of the revised convergence insufficiency symptom survey in adults. Ophthalmic Physiol. Opt..

[4] Simon J., Buckley E., Drack A., Hutchinson A., Plager D., Raab E. (2006). Basic and Clinical Science Course.

[5] Coles-Brennan C., Sulley A., Young G. (2019). Management of digital eye strain. Clin. Exp. Optom..

[6] Khushdeep K., Harpinder K., Sidhu M. (2015). Computer vision syndrome: A major concern for VDT users. Asian J. Home Sci..

[7] Abuallut I., Ajeebi R.E., Bahari A.Y., Abudeyah M.A., Alyamani A.A., Zurayyir A.J., Alharbi A.H., Al Faqih A.A., Suwaydi A.Z., Alqasemi M.I. (2022). Prevalence of computer vision syndrome among school-age children during the COVID-19 pandemic, Saudi Arabia: A cross-sectional survey. Children.

[8] Song M., Li L., Guo J., Liu T., Li S., Wang Y., Ain Q.U., Wang J. (2020). A new method for muscular visual fatigue detection using electrooculogram. Biomed. Signal Process. Control.

[9] Chin B., Faibish B., Hisaka C., Thal L., Tsuda K. (1995). A survey of the treatment of convergence insufficiency by optometrists in the greater San Francisco Bay area. J. Behav. Optom..

[10] Scheiman M., Cooper J., Mitchell G.L., De Land P.A., Cotter S., Borsting E., London R., Rouse M. (2002). A survey of treatment modalities for convergence insufficiency. Optom. Vis. Sci..

[11] Scheiman M., Mitchell G.L., Cotter S., Cooper J., Kulp M., Rouse M., Borsting E., London R., Wensveen J., Convergence Insufficiency Treatment Trial (CITT) Study Group (2005). A randomized clinical trial of treatments for convergence insufficiency in children. Arch. Ophthalmol..

[12] Gallaway M., Scheiman M., Malhotra K. (2002). The effectiveness of pencil pushups treatment for convergence insufficiency: A pilot study. Optom. Vis. Sci..

[13] (2008). Convergence Insufficiency Treatment Trial Study Group. Randomized clinical trial of treatments for symptomatic convergence insufficiency in children. Arch. Ophthalmol..

[14] Scheiman M., Kulp M., Cotter S., Mitchell G.L., Rouse M., Hertle R., Cooper J., Coulter R., Gallaway M., Hopkins K. (2009). Long-term effectiveness of treatments for symptomatic convergence insufficiency in children. Optom. Vis. Sci..

[15] Li T., Li C., Zhang X., Liang W., Chen Y., Ye Y., Lin H. (2021). Augmented reality in ophthalmology: Applications and challenges. Front. Med..

[16] Li S., Tang A., Yang B., Wang J., Liu L. (2022). Virtual reality-based vision therapy versus OBVAT in the treatment of convergence insufficiency, accommodative dysfunction: A pilot randomized controlled trial. BMC Ophthalmol..

[17] Islam T., Roy A.D. (2025). A virtual approach: Systematic review and meta-analysis of virtual reality-based therapies for convergence insufficiency. J. Optom..

[18] Ward L.M., Gaertner C., Olivier L., Ajrezo L., Kapoula Z. (2020). Vergence and accommodation disorders in children with vertigo: A need for evidence-based diagnosis. eClinicalMedicine.

[19] Badiali G., Cercenelli L., Battaglia S., Marcelli E., Marchetti C., Ferrari V., Cutolo F. (2020). Review on augmented reality in oral and cranio-maxillofacial surgery: Toward “surgery-specific” head-up displays. IEEE Access.

[20] Raghuram A., Cotter S.A., Gowrisankaran S., Kanji J., Howell D.R., Meehan W.P., Shah A.S. (2019). Postconcussion: Receded near point of convergence is not diagnostic of convergence insufficiency. Am. J. Ophthalmol..

[21] Goh C., Puah M., Toh Z.H., Boon J., Boey D., Tay R., Sule A.A., Liu R., Ong X.E., Kalra A. (2025). Mobile Apps and Visual Function Assessment: A Comprehensive Review of the Latest Advancements. Ophthalmol. Ther..

[22] Nagino K., Sung J., Midorikawa-Inomata A., Eguchi A., Fujimoto K., Okumura Y., Miura M., Yee A., Hurramhon S., Fujio K. (2024). Clinical utility of smartphone applications in ophthalmology: A systematic review. Ophthalmol. Sci..

[23] Subbiah A., Aik K.L.T., Kumar B.K. (2025). Mobile Health Diagnostics for Accommodative Dysfunction: Leveraging Handheld Device Technology for Near Point of Convergence Testing. IEEE Int. Colloq. Signal Process. Its Appl. (CSPA).

[24] Khatib A., Raz S., Nasser H., Jabaly-Habib H., Shimshoni I. (2024). AI-powered smartphone diagnostics for convergence insufficiency. J. Clin. Transl. Ophthalmol..

[25] Khatib A., Jabaly-Habib H., Raz S., Shimshoni I. (2026). Enhancing Ophthalmologists’ Accuracy in Detecting Convergence Insufficiency Using AI-Derived Graphical Outputs. J. Clin. Transl. Ophthalmol..

[26] Korot E., Pontikos N., Drawnel F.M., Jaber A., Fu D.J., Zhang G., Miranda M.A., Liefers B., Glinton S., Wagner S.K. (2022). Enablers and barriers to deployment of smartphone-based home vision monitoring in clinical practice settings. JAMA Ophthalmol..

[27] Sharma M., Jain N., Ranganathan S., Sharma N., Honavar S.G., Sharma N., Sachdev M.S. (2020). Tele-ophthalmology: Need of the hour. Indian J. Ophthalmol..

[28] Woodward M.A., Ple-Plakon P., Blachley T., Musch D.C., Newman-Casey P.A., De Lott L.B., Lee P.P. (2015). Eye care providers’ attitudes towards tele-ophthalmology. Telemed. e-Health.

[29] Nikolaidou A., Tsaousis K.T. (2021). Teleophthalmology and artificial intelligence as game changers in ophthalmic care after the COVID-19 pandemic. Cureus.

[30] Vakunov A., Lagun D. (2020). MediaPipe Iris: Real-time iris tracking and depth estimation. Google AI Blog.

[31] Hashemi H., Khabazkhoob M., Emamian M.H., Shariati M., Yekta A., Fotouhi A. (2015). White-to-white corneal diameter distribution in an adult population. J. Curr. Ophthalmol..

[32] Bergmanson J.P., Martinez J.G. (2017). Size does matter: What is the corneo-limbal diameter?. Clin. Exp. Optom..

[33] Gharaee H., Abrishami M., Shafiee M., Ehsaei A. (2014). White-to-white corneal diameter: Normal values in a healthy Iranian population obtained with the Orbscan II. Int. J. Ophthalmol..

